# Dazzled by the Mystery of Mentalism: The Cognitive Neuroscience of Mental Athletes

**DOI:** 10.3389/fnhum.2017.00287

**Published:** 2017-05-31

**Authors:** Andres Rieznik, Mikhail Lebedev, Mariano Sigman

**Affiliations:** ^1^CONICET Buenos Aires, Argentina; ^2^El Gato y La Caja Buenos Aires, Argentina; ^3^Neuroscience Laboratory, Universidad Torcuato Di Tella Buenos Aires, Argentina; ^4^Center for Neuroengineering, Duke University Durham, NC, United States

**Keywords:** mental athletes, augmented cognition, prodigies, savant syndrome, arithmetic operation

Neural processing by mental athletes (MAs) has received attention from Neuroscience community, with several publications examining superior memorizers (Maguire et al., 2003; Bor et al., 2008), lighting calculators (Pesenti et al., 2001; Fehr et al., 2010), and savants (Treffert, 2009). In this opinion, we contend that the presumption of extraordinary abilities in MAs is fundamentally flawed because their demonstrations involve tricks that regular individuals can learn. Since, these tricks easily escape the scrutiny of investigators, a high standard of rigor should be applied to research on MAs.

MAs seem to demonstrate abilities—for example, short-term memory and mental calculations—that by far exceed those of an average person. MAs' performance is indeed impressive: for instance, some of them multiply two 20-digits numbers without annotating and the other memorize thousands of digits of π.

Yet, such demonstrations utilize tricks that are not apparent to the public but well-known to magicians. Using these tricks, virtually any person can reach the level of MA performance with some practice (Benjamin and Shermer, 2008; Doerfler, 2008); no extraordinary brain is required. Indeed, there is no structural differences between the brains of superior memorizers and control subjects (Maguire et al., 2003), and the total time of deliberate practice is the best predictor of chess prodigies' performance (Ericsson and Charness, 1994).

We do not deny here that innate factors contribute to mathematical abilities (Docherty et al., 2010), but argue that cognitive science of extraordinary mental skills should not be based on anecdotes and ambiguous claims lacking rigorous proofs. We contend that scientists should be highly skeptical about the tempting assumption that extraordinary performance of MAs stems from a natural and unique gift (Murray, 1989).

A cognitive study would typically examine a MA performing an exceptionally difficult calculation, but neglect the fact that the calculation could be based on a simplifying algorithm. For example, in a recent study (Fehr et al., 2010) a prodigy reported the third and fourth digits for the exponentials, x^y^. To a trained magician this immediately suggests a trick. For example, the task can be simplified by memorizing a table containing 990 two-digit numbers (90 possible bases times 11 possible exponents). This memorization requires substantial training using standard memorizing techniques but is still much easier than the full calculation.

The same prodigy recently participated in a study where he calculated the sine of an angle, given in degrees, up to 10-digit precision (Pesenti et al., 2001). Although there is no well-known mental algorithm for this calculation, a memorization strategy was likely involved. It is possible that the task was simplified by memorizing a table containing 91 ten-digit numbers (one number for each angle between 0 and 90°) and using trigonometric relations, such as sin(φ) = sin(φ + 180), for the angles outside from the 0 to 90° range.

In these examples, an introspective report in which the prodigy explains and describes his calculations would have been of great value from both the algorithmic viewpoint and the evaluation of mental processes involved. Such, a report could be also helpful to gain an understanding of how complex operations are parsed into solvable mental algorithms, the parcellation that can be analyzed using response times for different input and output numbers, sequences and digits (Sigman and Dehaene, 2005). Overall, a rigorous analysis of the mental strategy is highly desirable, including all possible shortcuts and tricks. This information is key to the understanding of cognitive skills of MAs.

Currently, there are several famous MAs who are candidates for a scientific scrutiny. Daniel Tammet is famous because of his extraordinary calculation abilities. He is attributes his skills to an epileptic attack at his infancy (Foer, 2011, Chapter 10, p. 215). Although the connection between epileptic attacks and mental abilities could be of interest, several skepticism has been raised concerning Tammet's case (Doerfler, 2008; Foer, 2011). In his best-selling book “*Moonwalking with Einstein*,” Joshua Foer suggested that Tammet's achievements could be the result of extensive deliberate practice rather than a feature of his exceptional mind. Curiously, The New York Times review of Foer's book (Horowitz, 2011), raised a criticism because “Foer inexplicably devotes space to attempting to convince the reader that Daniel Tammet, a renowned savant who memorized 22,514 digits of pi, may not actually be doing it naturally.” We certainly disagree with this criticism and argue that an examination of mental strategy should be the crucial issue in cognitive studies of MAs. The entire point of investigating prodigies is to decipher the cognitive mechanisms by which they perform their feats. If the magic tricks remain hidden there is very little that can be done about such cases scientifically.

In Tammet's case, an evidence of a memorization strategy has been obtained by Ronald Doerfler who investigated Tammet's response times while mentally dividing 13 by 97 (Doerfler, 2008). This particular operation is well-known to MAs because dividing by 97 results in a repetition of shifted patterns of 96 digits and hence can be remembered. The hypothesis that a MA uses this algorithm has a concrete prediction: he should slow down during the transitions between these remembered chunks. This is exactly what Doerfler observed when measuring the speed of recitation in Tammet's calculation. Doerfler concluded that a classic mnemonic technique was much more likely than more glamorous explanations such as the formation of synesthetic landscapes for the representation of numbers (Doerfler, 2008).

Notably, both Doerfler and Foer are MAs themselves, so they have a good understanding of the conjuring that tricks the audience, including the scientists investigating MAs.

Several myths about autistic savant calendrical calculators have been debunked by Cowan and Frith (2009). Calendrical calculation is a paradigmatic example of mentalist demonstration because it has the virtue to appear much more difficult than it really is (Benjamin and Shermer, 2008).

Despite these critical publications, many scientific publications state that an individual is a savant without presenting substantial proof. For instance, Darold Treffert gave the following description of an artistic savant (Treffert, 2009): “Stephen Wiltshire can certainly replicate in stunning fashion what he sees as demonstrated in a recent documentary film clip, when, after a 45 min helicopter ride over Rome, he completed, in a 3-day drawing marathon, an impeccably accurate drawing, on a five and half yard canvas. It captures with precision the many square miles he has seen street by street, building by building and column by column.” To our knowledge, this depiction has not been confirmed or quantified by any real study.

In summary, we have pointed out several issues regarding research on prodigious and exceptional performers:

1. Overreliance on anecdotal and unreproducible individual cases.
2. Methodological errors in the evaluation of MA performance, which make the performance appear more exceptional than it really is.
3. Ignorance about techniques employed by performers to achieve their feats. These techniques involve training but do not require extraordinary mental abilities.

The third issue is particularly important for the understanding of MAs' motivation. Why do MAs prefer to show themselves as virtuosos rather than the product of tremendous effort and practice? The reason is simple: even young children understand that calculations are more impressive if they do not rely on standard techniques like using their fingers. Mental calculations appear more remarkable if they mysteriously pop-out in the air from unexplained virtues of an unexplained brain.

The task of cognitive neuroscience is to unveil the real technique behind the seemingly extraordinary performance. A healthy skepticism can prevent scientists from making misleading conclusions when dazzled by the magic of extraordinary performers. We believe that this note may be especially timely now, when the validity of psychological and cognitive studies is being questioned and discussed (Open Science Collaboration, 2015).

While this article was under review, Dresler et al. published a study (Dresler et al., 2017), where they compared fMRI patterns in mental athletes vs. normal subjects trained, reaching the conclusions that are similar to the ones expressed in this opinion article. They found mnemonic training in regular subjects induces functional connectivity changes that were similar to the ones observed in memory champions. In agreement with our position, they concluded that “mnemonic strategies for superior memory can be learned by naive subjects.” Had the scientific community a better knowledge of typical MAs' procedures, such a conclusion would be rather obvious. In many cases, experiments would not be even needed to prove that anyone can learn a technique. (For example, experiments are hardly needed to show that regular people can learn to bend a spoon using Uri Geller's techniques, and the power of mind is not required for such performance.) We are pleased by the fact that the study of Dresler et al. represents a step forward toward a more rigorous, mysticism-free cognitive neuroscience of prodigies.

## Author contributions

All authors listed, have made substantial, direct, and intellectual contribution to the work, and approved it for publication.

### Conflict of interest statement

The authors declare that the research was conducted in the absence of any commercial or financial relationships that could be construed as a potential conflict of interest.
