# Corrigendum: Dazzled by the mystery of mentalism: the cognitive neuroscience of mental athletes

**DOI:** 10.3389/fnhum.2025.1569293

**Published:** 2025-03-13

**Authors:** Andres Rieznik, Mikhail Lebedev, Mariano Sigman

**Affiliations:** ^1^CONICET, Buenos Aires, Argentina; ^2^El Gato y La Caja, Buenos Aires, Argentina; ^3^Neuroscience Laboratory, Universidad Torcuato Di Tella, Buenos Aires, Argentina; ^4^Center for Neuroengineering, Duke University, Durham, NC, United States

**Keywords:** mental athletes, augmented cognition, prodigies, savant syndrome, arithmetic operation

In the published article, in eighth paragraph, the following text: “Daniel Tammet is famous because of his extraordinary calculation abilities. He is attributes his skills to an epileptic attack at his infancy,” was incorrectly attributed to: Bor et al., 2008. It should be attributed to: Foer, 2011, Chapter 10, p. 215.

The authors apologize for this error and state that this does not change the scientific conclusions of the article in any way. The original article has been updated.

